# Reading and math anxiety in children: differential roles of state and trait components in academic performance, and the moderating effects of intelligence and time pressure

**DOI:** 10.3389/frcha.2026.1778068

**Published:** 2026-05-08

**Authors:** Lars Orbach, Florian Schmitz, Annemarie Fritz, Klaus Willmes

**Affiliations:** 1 Academy Word and Number, Cologne, Germany; 2 Department of Psychology, University of Duisburg-Essen, Essen, Germany; 3 Section Neuropsychology, Department of Neurology, University Hospital Aachen, RWTH Aachen University, Aachen, Germany

**Keywords:** academic performance, math anxiety, primary school children, reading anxiety, state-trait-anxiety

## Abstract

In the last decades, reading anxiety (RA) has received considerably less attention than math anxiety (MA), and its conceptualization remains underdeveloped. Building on the state-trait anxiety framework, the present study examines state and trait components of RA and MA and their relations to academic performance under conditions with and without time pressure. A sample of 311 third- and fourth-grade students (51.4% girls) completed domain-specific state and trait anxiety questionnaires, reading and math achievement tests administered under timed and untimed conditions, and a measure of fluid intelligence. State anxiety was assessed before and after performance tests as well as in a reference condition to capture situational changes. Results indicated that RA, like MA, represents a hierarchical construct with distinct but related state and trait components and clear evidence for domain specificity. Time pressure increased state anxiety in both reading and math, where children with higher baseline state anxiety showed reduced situational adjustment. Anxiety-performance relations differed across domains. Reading performance was negatively associated with trait RA, baseline state RA, and anticipatory state RA under time pressure, whereas no such relations emerged in the untimed condition. In contrast, math performance was consistently negatively related to trait and baseline state MA across both testing conditions. Fluid intelligence emerged as the strongest predictor of performance, with only limited evidence for moderation by anxiety. Overall, these findings highlight the importance of distinguishing between state and trait components of domain-specific anxiety and demonstrate that situational demands, such as time pressure, play a critical role in shaping anxiety-performance relations in children.

## 1 Introduction

Anxiety is a fundamental human emotion that arises in response to perceived or anticipated threat and encompasses emotional, cognitive, physiological, and behavioral components (1). The expression and intensity vary across developmental stages, largely as a function of cognitive development and social experiences (2, 3). As children enter formal schooling, they encounter new context-specific stressors that may elicit anxiety responses (4). Research has shown that children are particularly vulnerable to anxieties related to academic performance and evaluation within educational environments. These anxieties are frequently intensified by mechanisms of social comparison and self-perceived competence (5, 6), which may undermine their capacity to engage effectively with instructional material and to fully benefit from educational contexts.

Math and reading are widely recognized as fundamental elements in the school education of children (7). While extensive research over the past two decades has systematically examined math-related anxieties (MA) in school-aged children (8), comparatively less attention has been devoted to the phenomenon of reading anxiety (RA) (9). Since the late 1950s, an increasing number of studies have focused on MA and its detrimental effects on the development of math skills and situational math performance, initially concentrating on young adults and, since the millennium, increasingly addressing school-aged children (10, 11). Robust evidence has established the negative impact of MA, highlighting its complex interactions with cognitive processes and academic outcomes (12–14). In contrast, research addressing anxiety in reading contexts remains scarce, with neither clear empirical consensus nor a well-defined international research community dedicated to the study of RA (9, 15). Consequently, the handful of studies on RA often adapted self-report questionnaires from MA research (15–17), which carries the risk of repeating previous problems associated with differences in item contents and latent structures. Over the past two decades, research on math anxiety (MA) in children has expanded considerably, paralleled by the development of numerous questionnaires grounded in diverse theoretical frameworks and employing varying item formats (8, 18). This proliferation of instruments has contributed to inconsistent findings across studies, suggesting that variations in content and latent structures of these measures may underlie the observed discrepancies (19, 20).

The present study aims to enhance the conceptualization and assessment of RA by adapting Spielberger's (21) state-trait anxiety model. The model distinguishes between anxiety as a state and as a personality trait. State anxiety is characterized by a temporary anxiety reaction involving an increased arousal of the autonomic nervous system, whereas trait anxiety reflects a relatively enduring individual disposition towards anxiety. More broadly, anxiety is often conceptualized as a hierarchical construct, in which a broader higher-order factor represents a general vulnerability to experiencing anxiety, while more specific low-order factors represent distinct manifestations of anxiety. Within this framework, different anxiety symptoms share a common underlying component of negative affect or general distress, yet also reflect more narrowly defined fears associated with particular situations (22–26). In the academic settings, these lower-order dimensions may include domain specific forms of anxiety, such as RA and MA. Addressing a current gap in the literature, the present study directly compares RA and MA within a single sample. Specifically, it aims to examine differences between the two constructs and to develop a valid instrument for assessing trait-RA. Additionally, the study investigates the moderating roles of time pressure and intelligence to better understand their impact on the experience and expression of RA.

### 1.1 Previous findings on MA

Intensified research on MA in school-aged children has yielded insights into how MA affects children's number manipulation and contributes to deficits in numerical reasoning (8, 11). Studies have identified MA as early as kindergarten and in the first grades of elementary school (27–29). Numerous findings indicate small to moderate correlations between MA and math fluency (12–14). The association between anxiety and math fluency is commonly attributed to disruptions in the attention control system and to avoidance behavior (30). According to the Attentional Control Theory [ACT; (31)], individuals experiencing anxiety during task processing have portions of their working memory resources blocked by anxiety-related thoughts, which in turn impairs problem-solving in math. Consequently, their attentional focus shifts from task-oriented processing to threat-related stimuli (e.g., worrisome thoughts or external stimuli). In the context of math, this mechanism has been proposed to explain the association between MA and reduced math fluency. Because individuals tend to avoid aversive stimuli, children with higher levels of anxiety may enter a vicious cycle in which reduced engagement with the respective domain limits opportunities to build proficiency (32–34). Interestingly, MA shows a unique phenomenology that cannot be explained by other types of anxiety. Research has found that the connection between MA and math performance cannot be explained by social-, test-, or general anxiety (10, 35–39), and has identified specific genetic and environmental factors that contribute to MA (40–43).

In recent years, questions have been raised regarding the factorial structures of MA questionnaires for children, as research on school-aged children has revealed some discrepancies compared to studies on adults (19, 44). Meta-analyses examining the relationship between MA and math fluency in children further emphasize that the choice of MA questionnaire can influence the study outcomes (12–14). While research conducted on adults has predominantly employed the Mathematics Anxiety Rating Scale (MARS) or shorter versions (AMAS; sMARS; MARS-R), intensified research on MA in children has resulted in the development of a variety of age-appropriate questionnaires (10, 18). It has therefore been suggested that some of the discrepancies across studies may arise from differences in the latent structures of these instruments, reflecting a lack of comparability. Recent studies have provided evidence to support this hypothesis (20, 44).

**Figure 1:**
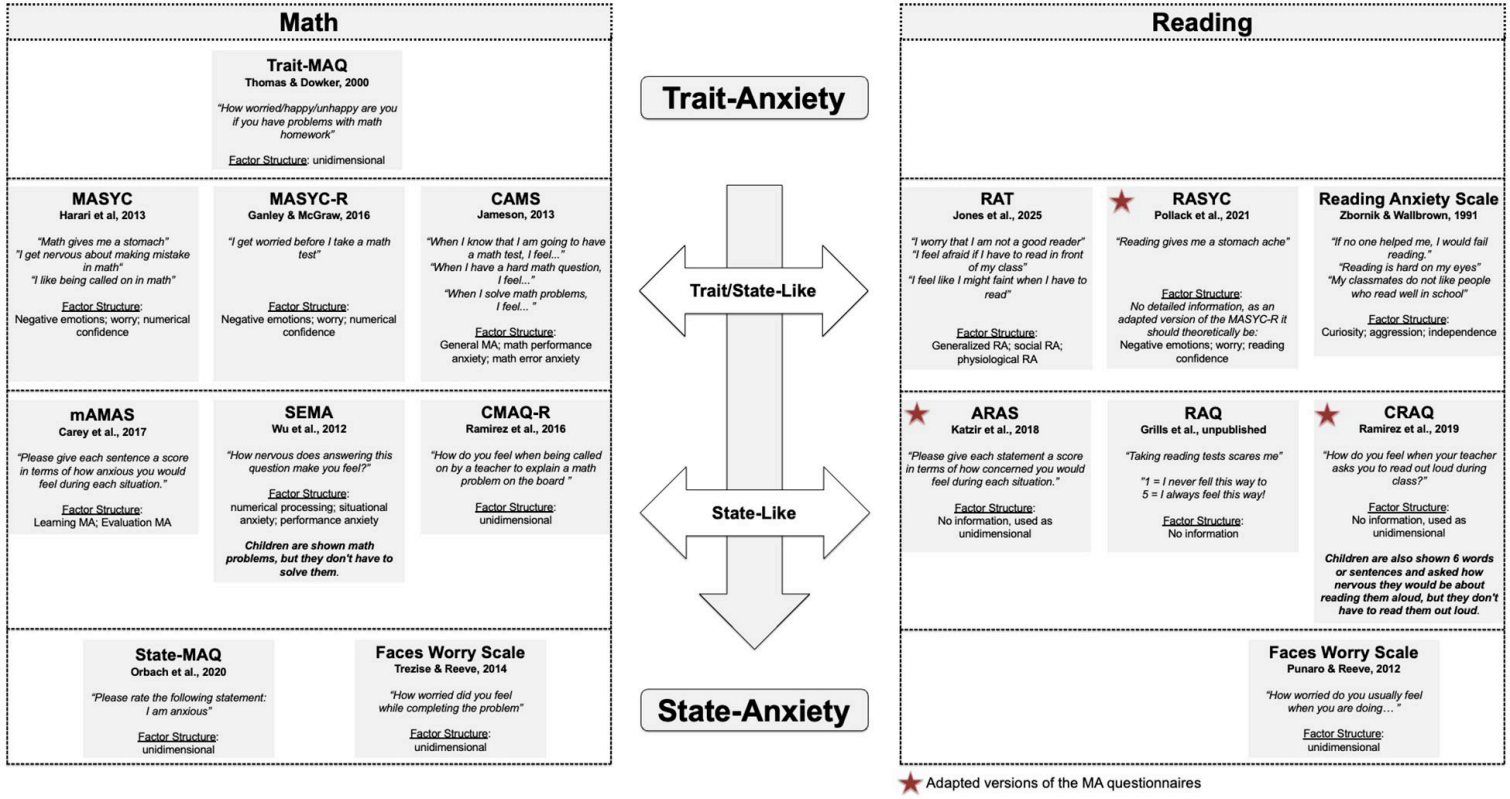
MA and RA questionnaires in young children.

Studies investigating the impact or intensity bias in MA have emphasized the state-trait distinction as an important criterion for classifying MA questionnaires. Research indicates that retrospective or hypothetical evaluations may capture subjective beliefs rather than actual emotional states, highlighting differences between state and trait assessments of anxiety (45–50). In line with Spielberger's state-trait model (21), assessing both state and trait components is particularly valuable: state anxiety represents a temporary, situational response accompanied by autonomic arousal, whereas trait anxiety reflects a relatively stable individual tendency to experience anxiety. Within this framework, current MA questionnaires can be categorized (Figure 1) according to whether they primarily assess state or trait aspects, offering a more nuanced understanding of the specific dimension of anxiety captured by each instrument. Distinguishing between these components enhances conceptual clarity by separating situational anxiety reactions from enduring dispositions and helps integrate research on stable cognitive predictors of future behavior (47) with studies exploring the interplay between state anxiety and cognitive processes during math task processing (51), thereby contributing to a more comprehensive understanding of MA.

Beyond examining the general relationship between MA and math fluency, recent research has increasingly shifted toward exploring the interplay how MA interacts with underlying cognitive processes (52), particularly working memory (WM) and executive functions (EF). In this context, potential moderation effects of these cognitive processes on the MA-performance link have been investigated. However, considerably less attention has been given to the relationship between MA and intelligence. While several studies have reported no relationship between intelligence and trait dispositions of MA (53, 54), one study identified a small but significant correlation between intelligence and state MA (10).

Another relatively underexplored topic in MA research is the influence of time pressure. While it is assumed that time pressure increases MA, few studies provide detailed insights into how this interplay affects children (55). This is noteworthy, as both time pressure (56, 57) and MA (58, 59) appear to influence the selection of problem-solving strategies. Research indicates that under time pressure, children exhibit higher levels of MA and tend to rely on faster but less accurate strategies, whereas longer time windows allow for more precise, deliberate strategy use (56, 57). These findings suggest that the combination of time pressure and MA may push children toward suboptimal strategies as part of a coping process.

### 1.2 Previous findings on RA

In contrast to the extensive research examining the predictive role of linguistic and cognitive factors on reading fluency in children, there has been a paucity of investigations into the role of RA (16, 60). Only recently, few studies have focused on the specific fear of reading (61). Earlier research investigated the impact of general anxiety (GA) on children's reading performance, with evidence suggesting that higher levels of GA are associated with poorer reading skills (17). A meta-analysis (62) implies that individuals with reading difficulties exhibit an intermediate risk for developing internalizing problems. This risk appears to be primarily linked to a higher probability of anxiety than of depression.

Although the association between GA and RA is well recognized, emerging evidence suggests that these two constructs may be separable (9, 63) and may be related to reading fluency in different ways (61, 64). In line with the definition of MA, RA can be conceptualized as a specific phobia of reading-related stimuli that elicit fear responses at the affective, physiological, cognitive, and behavioral levels (9, 60, 63, 65). It is hypothesized that this specific fear response may result in weaker reading fluency, due to cognitive interference, as proposed by ACT, and due to long term negative consequences such as avoidance behavior (16, 65).

However, there is a lack of research that investigated the relationship between RA and reading fluency in children (9, 17). This is also attributable to the limited number of available assessments for RA in children. Only in the past few years has a limited number of studies been published, most of which have adapted established MA questionnaires for use in reading contexts (see Figure 1). Unfortunately, there is often insufficient data available on the validity and reliability of these assessments. The adaptions of the MA self-reports AMAS, MAYSC-R and CMAQ represent a mixture of trait and state components, rendering them susceptible to the impact bias. Another questionnaire, the RAS (63), was developed on the basis of psychoanalytic case studies and includes scales that reflect psychoanalytic concepts such as aggression or curiosity (for example, “nasty children should fail reading”). More recently, a questionnaire [RAT (66)] was introduced to assess RA, including both self-report and parent-report versions and capturing a mixture of trait and state components. Most studies have indicated a significant negative correlation between RA and reading performance [(63): *r* = −0.4, RAS; (16): *r* = −0.28, ARAS; (15): *r* = −0.4, CRAQ; Piccolo *et al*., 2017: *r* = −0.32 to –0.40, RAS; (17): *r* = −0.4, RASYC; (64): r = −0.17 to −0.25, RAQ]. The remaining studies yielded correlations that were either partly significant [(61): *r* = −0.02 to −0.11, RAQ; (9): *r* = −0.22 to −0.30, CRAQ] or failed to identify a link between RA and reading performance [(67), Faces Worry Scale].

The extant research does not sufficiently address the distinctiveness of RA and MA, nor their interrelationships with domain-specific academic performances. The question of whether academic anxieties can be classified as a domain-specific fear reaction is currently debated (9). Only few studies have assessed domain-specific anxieties in math and reading (9, 17, 60), but they employed self-report measures without real-time assessments (state anxiety). The results indicate a moderate correlation between MA and RA questionnaires (*r* = 0.44–0.60). Sasanguie et al. (9) were able to identify discrete MA and RA factors through the application of a CFA in a sample of fifth graders. However, the lack of data on reading fluency precluded the researchers from establishing a link between the domain-specific anxieties and academic performance. Pollack et al. (17) identified that RA predicted math performance in students aged 8–13 years, while MA did not predict reading performance. In a study by Hill et al. (68), no association was identified between MA and reading performance in primary and secondary school children when the relation was controlled for GA. Considering these scarce findings, it may be hypothesized that there are separable constructs with domain-specific relationships to academic performance. Further research is needed to corroborate these assumptions. So far, research on RA has relied exclusively on questionnaire-based self-report measures, and neither situation-specific state anxiety questionnaires nor physiological correlates during reading tasks have been examined. In contrast, although research on MA has also predominantly relied on self-report questionnaires, a small number of studies have used physiological measures, and more recent work has increasingly incorporated state anxiety assessments (10, 30). Furthermore, no instrument is currently available for assessing trait RA, which precludes an analysis of the results in terms of state-trait differentiation.

### 1.3 Research aims of the present research

Compared to the extensive research findings on MA, studies on RA in children are rare, and both the conceptualization and assessment of RA are still evolving. Addressing this gap, the present study aims to advance the understanding of RA by assessing both math and reading anxieties through instruments that capture state and trait components. The study pursued three main research aims.

#### 1.3.1 Aim 1: Psychometrics and structure of anxiety measures

This study evaluated the psychometric characteristics of a newly developed trait questionnaire for RA (trait-RAQ) alongside other anxiety measures, namely state and trait anxiety in the domains of reading and math. Given that anxiety can be conceptualized as a hierarchical construct, we also examined how these different forms of anxiety cluster.

#### 1.3.2 Aim 2: State characteristics of reading and math anxiety and correlates

We examined the extent to which state anxiety increases in a test situation compared to a reference condition in which participants do not anticipate a test. We also tested whether individual differences in anxiety in the reference condition and changes in anxiety during a test situation are differentially related to trait anxiety. Further, we investigated the extent to which state and trait reading and math anxiety are associated with performance on corresponding ability tests. We predicted that test anxiety would be more strongly related to decreases in test performance when tests are administered under time pressure than when administered without time pressure.

#### 1.3.3 Aim 3: Testing moderation effects of test anxiety

The study examined test performance is jointly predicted by cognitive ability (positive), domain-specific anxiety (negative), and the interaction of both variables (i.e., whether the positive effect of ability is attenuated by anxiety).

## 2 Material and methods

### 2.1 Participants and procedure

The sample consisted of 311 third and fourth graders (51.4% girls; 120.03 ± 6.53 months old) from five schools in the state of North Rhine-Westphalia, Germany. Trained graduate students assessed entire classes within the schools during regular school lessons. All participants attended regular comprehensive schools and had no identified intellectual, communication, or language disorders. The data were collected on two consecutive days, with the survey taking place in 2-hour sessions. On the first day, state anxiety was assessed immediately prior to and after the timed reading comprehension test. The children then completed the reading comprehension test without time constraints and reported their state anxiety afterwards. After a short break, the children were asked to complete the trait-MA questionnaire and finally the intelligence test was administered. State-anxiety was also measured before and after the intelligence test. On the second day, participants filled out the state-anxiety questionnaire immediately prior to and after the timed math test. Afterwards, the children completed the math test without time constraints and reported their state anxiety afterwards. Finally, the children completed the trait-RA questionnaire.

Prior to completing the state anxiety questionnaire, children were informed that they would be taking a math, a reading test or a test with logical problems (intelligence test) and were shown a booklet containing various math/reading/logical problems. They were instructed to respond to the items specifically regarding the upcoming (or just completed) test situation. Other circumstances or general feelings were to be disregarded.

### 2.2 Materials

#### 2.2.1 State anxiety

State anxiety was measured using the State-Anxiety Questionnaire, a self-report measure consisting of seven items (instruction: “Please indicate to what extent each of the following statements applies to you in this moment. Please only give answers in accordance with your feelings and thoughts regarding the upcoming/completed math test.”; example items: “I am tense”, “I am nervous”, “I am worried”, “I am thinking about what might go wrong”, “I am restless”, “I am impatient”, “I am anxious”). Children were asked to indicate the extent to which each emotional state applied to them in the current situation, using a 4-point Likert scale ranging from 0 (“not at all”) to 3 (“very much”). The questionnaire is based on the State-Trait Anxiety Inventory [STAI; (69)] and was adapted using simple, brief, and age-appropriate language suitable for young children. The internal consistency of the state-MAQ was acceptable to good, with Cronbach's alpha values of *α* = .83 in the normative sample.

#### 2.2.2 Trait MA

A modified version of the Mathematics Anxiety Questionnaire [MAQ; (70)], adapted by Orbach *et al*. (20), was used to assess children's anxiety related to failure in mathematical contexts. In line with the hypothesis proposed by Krinzinger et al. (28), this version focuses specifically on anxiety triggered by potential failure in math-related situations. The instrument consists of seven items (e.g., “How anxious are you if you have problems with math homework?”), each referring to the same scenarios as in the original MAQ. Children rated their anxiety about failure on a 5-point Likert scale ranging from 0 (“not anxious”) to 4 (“very anxious”). The modified MAQ showed excellent internal consistency, with Cronbach's alpha values of *α* = .88 in the normative sample.

#### 2.2.3 Trait RA

Trait-Reading Anxiety, as children's anxiety of failure in reading, was assessed with a self-developed questionnaire (trait-RAQ) based on the item structure of the trait-MAQ. The questionnaire consists of seven items, each referring to a different reading-related situation (How anxious are you if you have problems with: reading in general, reading an unknown word, reading a text in front of the class, reading an easy text, reading a difficult text, reading homework in German at school, and understanding a text you have read in class). Children rated their anxiety about failure on a 5-point Likert scale (0 not anxious to 4 very anxious). Higher values referred to greater intensity of trait-RA.

#### 2.2.4 Math achievement under time pressure

Four basic arithmetic operation subtests (addition, subtraction, add the missing number, number sequences) of the Heidelberger Rechentest [HRT; (71)] were used to assess math performance. Three subtests included 40 tasks (addition, subtraction, add the missing number) and the number sequence subtest had 20 tasks with increasing difficulty. The participants were instructed to solve as many tasks as possible within two minutes. The total score (max. 140 points) was calculated as the sum of all correctly solved items. The reliability (internal consistency) was *α* = 0.77–0.89 for the norm sample.

#### 2.2.5 Math achievement without time pressure

Math achievement without time pressure was assessed with subtests of the Lost in Math test (72). The inventory was designed to measure basic math skills in the domains of the part–part–whole concept, multiplication, division, and understanding of the place value system that children should have developed before entering secondary school according to the national curriculum for Germany. Tasks included, for example, decomposing numbers into parts, solving multiplication and division problems, and applying place-value knowledge to multi-digit numbers. In this study, the children were asked to solve 36 items. The total score (max. 36 points) was calculated as the sum of all correctly solved items. The reliability (internal consistency) was *α* = 0.87 for the norm sample.

#### 2.2.6 Reading achievement under time pressure

To assess reading achievement under timed conditions, the standardized reading comprehension test [ELFE: (73)] was used. The ELFE includes three subtests under timed conditions, which measured reading comprehension skills, reading fluency and reading accuracy on word, sentence, and text level. The first subtest focuses on reading abilities at the word level by asking the children to solve as many items as possible within three minutes. Four words and one picture were presented, and the children were told to mark the word, matching the presented picture (72 items/pictures). On the sentence level, children had to mark one out of five words that completed a sentence grammatically, syntactically, and semantically correct (28 items/sentences; three-minute time limit). The last subtest presented short texts, and the children had to answer one to three multiple choice questions (one out of four) about the text content (20 items/questions; seven-minute time limit). The reliability (internal consistency) was *α* = 0.92–0.97 for the norm sample.

#### 2.2.7 Reading achievement without time pressure

The Lesegeschwindigkeits- und -verständnistest [LGVT: (74)] was used to assess reading achievement without time pressure. For this purpose, the assessment situation of the LGVT was modified by presenting the children with a text section of the LGVT with 533 words, which they had to read silently without any time pressure. At 7 given places in the text, the children had to choose one of three words that best fits into the given text context. The LGVT was designed for young secondary school children, so the difficulty of the text can be considered as challenging. The total score was calculated from the sum of all correctly solved items. The reliability (internal consistency) was *α* = 0.84–0.87 for the norm sample.

#### 2.2.8 Fluid intelligence

To assess fluid intelligence, the German adaptation CFT 20-R (75) of the Culture Fair Intelligence Test (76) was used. The assessment is a nonverbal group test that measures fluid intelligence in four figural tasks (continuing logical progressions, classifications, matrices, topological conclusions). The reliability (internal consistency) was *α* = 0.92.

### 2.3 Data analysis

#### 2.3.1 General information and data preprocessing

All analyses were conducted using the statistical environment R version 4.5.2 [R (77)]. The package psych version 2.5.6 (78) was used for psychometric analyses, the package lavaan version 0.6–20 (79) and semTools version 0.5–7 (80) were employed for confirmatory factor analyses (CFA) and structural equation modeling (SEM). The package factoextra version 1.0.7 (81) was used for the hierarchical cluster analysis and dendextend version 1.19.1 (82) was applied for color coding of the clusters in the obtained dendrogram. The package bootnet version 1.6 (83) was employed to estimate Gaussian graphical models The package lm.beta version 1.7–3 (84) was used to derive standardized regression coefficients and the package rockchalk version 1.8.157 (85) was utilized to inspect moderation effects with help of Johnson-Neyman plots.

Data were checked for missing values on the level of individual values and scores, and participants with more than four missing scores for anxiety scales were excluded (inspection of missing values revealed that only few participants had an increased proportion of missings that could be excluded when using this cut; see below). Multivariate extreme values were checked by computing Mahalanobis D scores, and applying the strict Tukey criterion (i.e., exceeding 1.5 interquartile ranges on top of the 75 percentile of ascending values).

The reading and math performance tests yielded two raw indicators of test performance: one corresponding with the number of correctly solved items in the given time, the other with commission errors. As speed-accuracy trade-offs can occur between both raw indicators at any level of performance (i.e., fast but inaccurate or slow but accurate responding), we averaged both raw indicators with equal weights into an integrative performance score by computing the difference of standardized raw indicators (i.e., performance = z(correct)—z(errors)).

While CFA measurement models for each scale were assessed using the seven individual items as indicators, the comparatively complex latent change score models (see below) were estimated on the basis of 3 item parcels per measurement point. The latter was chosen to reduce model complexity and because we were interested in latent mean changes and relations of the factors rather than the measurement models (95). To this end, we averaged two pairs of highly correlated (synonymous) state indicators (1,2 and 5,6) and the remaining three items (3,4,7). This yielded three parcels in each measurement model without residual correlations or inflated construct saturation. The same item numbers were aggregated into parcels for the trait scales, although item wording was different and no residual item correlations needed to be committed in the measurement models of trait anxiety.

As some of the items and scales were skewed to the right, a robust estimation procedure was used for the CFA, using maximum likelihood estimation with robust (Huber-White) standard errors (MLR estimator). Missing values were imputed using Full-Information-Maximum-Likelihood (FIML). Scaled fit indices were computed for the structural equation models (86). Model fit was evaluated based on several fit indices, applying commonly used benchmarks of satisfactory fit [RMSEA < .07; SRMR < .08; CFI, TLI > .90; e.g., (87)].

#### 2.3.2 Psychometrics and structure of anxiety measures (RQ 1)

Scale statistics were computed for the anxiety measures, including conventional descriptive statistics (M, SD), internal consistency (Cronbach's *α*; with 95% confidence interval) and factor saturation (McDonald's *ω*; with 95% bias-corrected and accelerated (BCa) bootstrap interval). CFA analyses were conducted to test the adequacy of the measurement models for each scale. In the state scales, residual correlations were tested and allowed for two pairs of items with synonymous wordings (“I am tense (1)” with “I am nervous (2)”; and “I am restless (5)” with “I am impatient (6)”). Uni-dimensionality was ascertained by means of CFA, specifying one latent factor that accounted for the shared variance in the seven items of each scale. Quintile plots were used to inspect differential stability and state-sensitivity of the anxiety state measures in terms of level (for more detailed analyses, in particular with respect to state-dependent variance, see RQ 2). The structure of anxiety measures was investigated by means of a hierarchical cluster analysis using Ward's D2 agglomerative method based on Person correlations of the scales. Further, the robustness of the scale associations was tested with help of Gaussian graphical models [GGM; (83)] using a graphical least absolute shrinkage and selection operator in combination with extended Bayesian information criterion [EBICglasso; (88)].

#### 2.3.3 State characteristics of reading and relations to test performance (RQ2)

As difference scores based on observed indicators suffer from compromised reliability (89), state-dependent changes in anxiety were estimated by means of latent change score (LCS) models (90). LCS models allow to estimate condition-specific changes in factor means as well as condition-specific variance relative to a baseline condition used as a reference.

In this study we specified the state anxiety at the end of the respective assessment session as a reference. We assumed that state anxiety would be at baseline again as participants knew that no further test was to be taken. By contrast, state anxiety experienced prior and post the test under time pressure was used as the conditions with predicted elevated levels, due to anticipated pressure (“How much anxiety do you currently experience [when anticipating the test]?”) and in retrospective evaluation (“How much anxiety did you experience during the test [just taken]?”). Measurement models comprised three parcels, and corresponding specificities were allowed to be correlated across time points (as exemplified for the first indicator in Figure 5). Strict measurement invariance across time points (equal loadings, intercepts, and residuals of corresponding indicators) was established.

**Figure 2:**
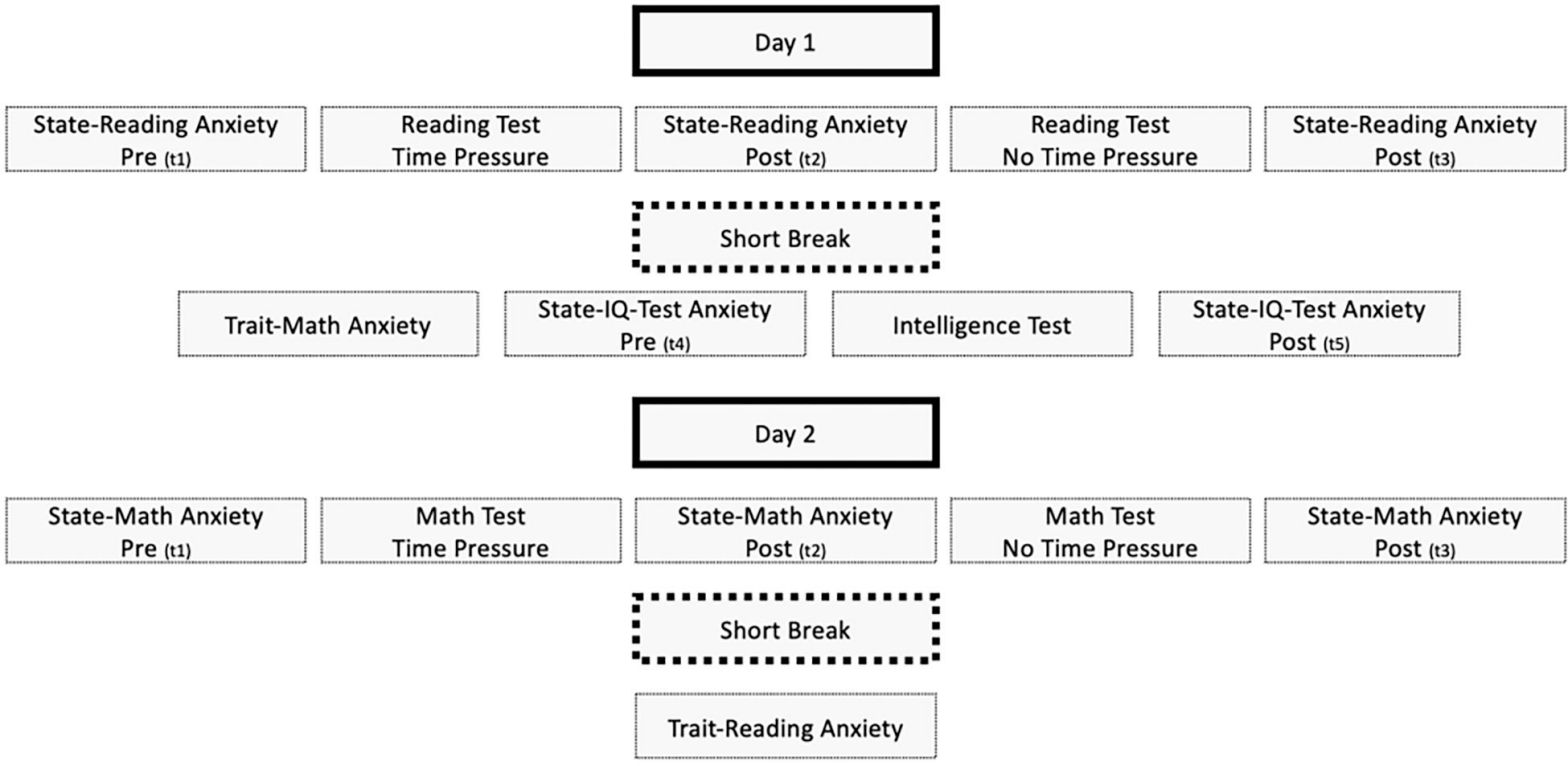
Research design.

Technically, the LCS model assesses changes in the time-pressure situations relative to the reference condition, and allows to estimate relative changes in level and (co-) variation of individual differences in experienced anxiety (see Figure 5). Trait anxiety and test performance in the respective domain were specified as correlates of level and change, the first as a latent factor based on three item parcels, the latter by entering the integrative performance scores.

#### 2.3.4 Testing moderation effects of test anxiety (RQ3)

Moderated regression analyses were specified for the four performance tests (i.e., in the domains reading and math, administered under time pressure and not). Separate regression models were specified for each anxiety scale (three states and one trait) to avoid problems associated to multi-collinearity of the anxiety scales. Thus, each regression model comprised as predictors: general cognitive ability, one of the anxiety scales and the interaction of both. The interaction term was computed based on centered constituent scores to reduce non-essential collinearity. In case of a significant interaction term, Johnson-Neyman plots were inspected to understand the moderation effects.

## 3 Results

### 3.1 Data preprocessing

On average 7.68% of all individual item data were missing, but there were few participants with a large proportion of missing values. After excluding participants with more than four missing scale scores (*n* = 22; average proportion of missing items: 63.59%), the remaining *n* = 289 participants had a proportion of 4.89% missing values, which appeared to be missing completely at random (Little's MCAR tests: $X^{2}(3,189)=\;2,267$, *p* = 1.00, 48 missing patterns, a non-parametric test using the MissMech R package version 1.0.4 yielded a p-value of .49, indicating no evidence of heteroscedasticity and no evidence that data are not MCAR). The check for multivariate outliers using Mahalanobis distances D scores did not indicate cases that exceeded the strict Tukey criterion.

#### 3.1.1 Psychometrics and structure of anxiety measures (RQ 1)

Descriptive scale statistics are presented in Table 1 for all anxiety scales. The mean item ratings were rather low on the respective scales (range: 0–4) for the reading anxiety state items (item means: 0.74–0.93), the math anxiety state items (item means: 0.72–0.94), and the IQ test anxiety state items (0.99; 1.00). They were only slightly higher for the two trait scales (reading trait anxiety: 1.48; math trait anxiety: 1.44) which used different item wordings. As predicted, mean scores of reading and math anxiety were higher for the situations surrounding the time-pressure test (state 1 and 2) as compared with the reference condition at the end of the session (state 3; for a formal test see analyses pertaining to RQ 2). The trait scores yielded higher mean ratings than the state scores, but these scales had totally different item wordings and cannot be compared for this reason. All scales, including the newly created trait reading anxiety scale, displayed satisfactory internal consistency and factor saturation. The range of bias-corrected bootstrap intervals suggested that un-corrected estimates may be slightly increased because of the right-skewed distributions, as is frequently observed in clinical data.

**Table 1:** Scale descriptives.

#	Scale	*M (SD)*	*α* [95% CI]	*ω* [95% BI]
1	Reading Anxiety Trait	10.33 (6.39)	.88 [.86;.90]	.92 [.85;.90]
2	Reading Anxiety State 1	6.48 (4.72)	.77 [.73;.81]	.85 [.70;.80]
3	Reading Anxiety State 2	5.76 (4.92)	.81 [.77;.84]	.88 [.72;.83]
4	Reading Anxiety State 3	5.18 (5.17)	.85 [.82;.87]	.91 [.78;.87]
5	Math Anxiety Trait	10.06 (7.63)	.92 [.90;.93]	.94 [.90;.94]
6	Math Anxiety State 1	6.58 (5.35)	.83 [.80;.86]	.88 [.78;.86]
7	Math Anxiety State 2	6.83 (6.09)	.88 [.85;.90]	.91 [.85;.90]
8	Math Anxiety State 3	5.03 (5.67)	.89 [.87;.91]	.93 [.82;.91]
9	IQ Test Anxiety State 1	6.91 (5.68)	.87 [.85;.90]	.92 [.83;.90]
10	IQ Test Anxiety State 2	6.99 (5.97)	.87 [.84;.89]	.92 [.81;.89]

All scales comprised of 7 items. α = Cronbach's alpha [95% CI, confidence interval], ω = McDonald's omega [95% BI = bias-corrected and accelerated (BCa) bootstrap interval].

The CFA analyses indicated that the communality of the items of the trait scales could be well explained by one factor with decent fit. Analogous models were acceptable for most of the state scales. However residual correlations were permitted for theoretical reasons and to improve model fit. Both residual correlations were supported by significant X^2^-differences tests and by model comparison indices AIC and BIC in the current sample, further, bootstrap analyses confirmed the robustness of findings across 1,000 resamples (see Supplements S1 for model fits and S2 for tests regarding the permitted error covariances).

**Figure 3:**
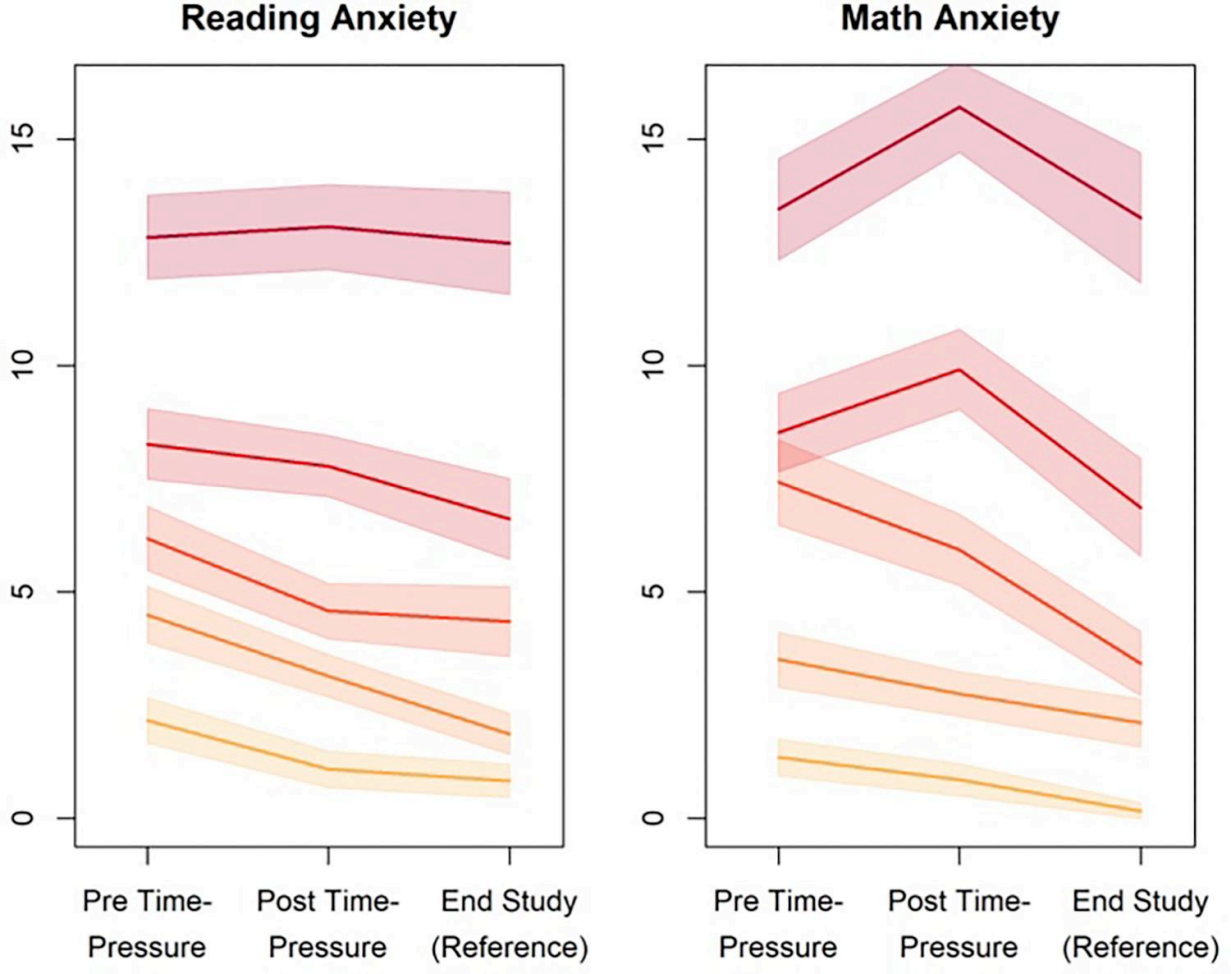
Levels of state anxiety. Anxiety scores across measurement points split by ascending quintiles of state anxiety; lines represent means with 95% CI.

A graphical inspection of state anxiety experienced across the three assessment conditions split by quintiles of the average state anxiety indicated both differential stability and predicted level changes (see Figure 3): The first could be inferred from that the order of the quintile trajectories was stable across measurement conditions, suggesting rank stability. The second could be inferred from the predicted general increase in level from the reference condition to the conditions surrounding the time-pressure performance test. It is of note that this increase appeared to be more pronounced for lower levels than for higher levels of experienced state anxiety, both in reading and in math anxiety. As discussed before, this attenuation of further increases for participants with generally high levels of anxiety cannot be solely attributed to ceiling effects of the scales (Figure 3 displays sum scores that have a theoretical range from 0 to 28 for the 7-item scales). A comprehensive formal test of these differential changes is provided by means of the LCS model (reported below).

Interestingly, there were hardly any gender differences in the reading anxiety scales. Female participants reported only descriptively increased reading anxiety trait (*d* = 0.22, *p* = .07) and state 1 (*d* = 0.19, *p* = .12) ratings, whereas the other state ratings did not differ (*d* ≤ .05, *p* ≥ .71). By contrast, math anxiety was moderately increased in female participants in the states surrounding the time-pressure math test (state 1: *d* = 0.31, *p* = .01; state 2: *d* = 0.27, *p* = .02), and tended to be increased also in the trait (*d* = 0.24, *p* = .05) and state 3 assessments (*d* = 0.24, *p* = .05). IQ test anxiety did not differ between both gender groups in any of the states (*d* ≤ .08, *p* ≥ .53). Objectively assessed reading performance did not differ substantially between girls and boys (*d* ≤ |.15|, *p* ≥ .21), whereas boys displayed moderately stronger test scores in the math tests (math with time pressure: *d* = 0.32, *p* = .01; math without pressure *d* = 0.43, *p* < .001). For details regarding gender differences, see Supplement S3.

**Figure 4:**
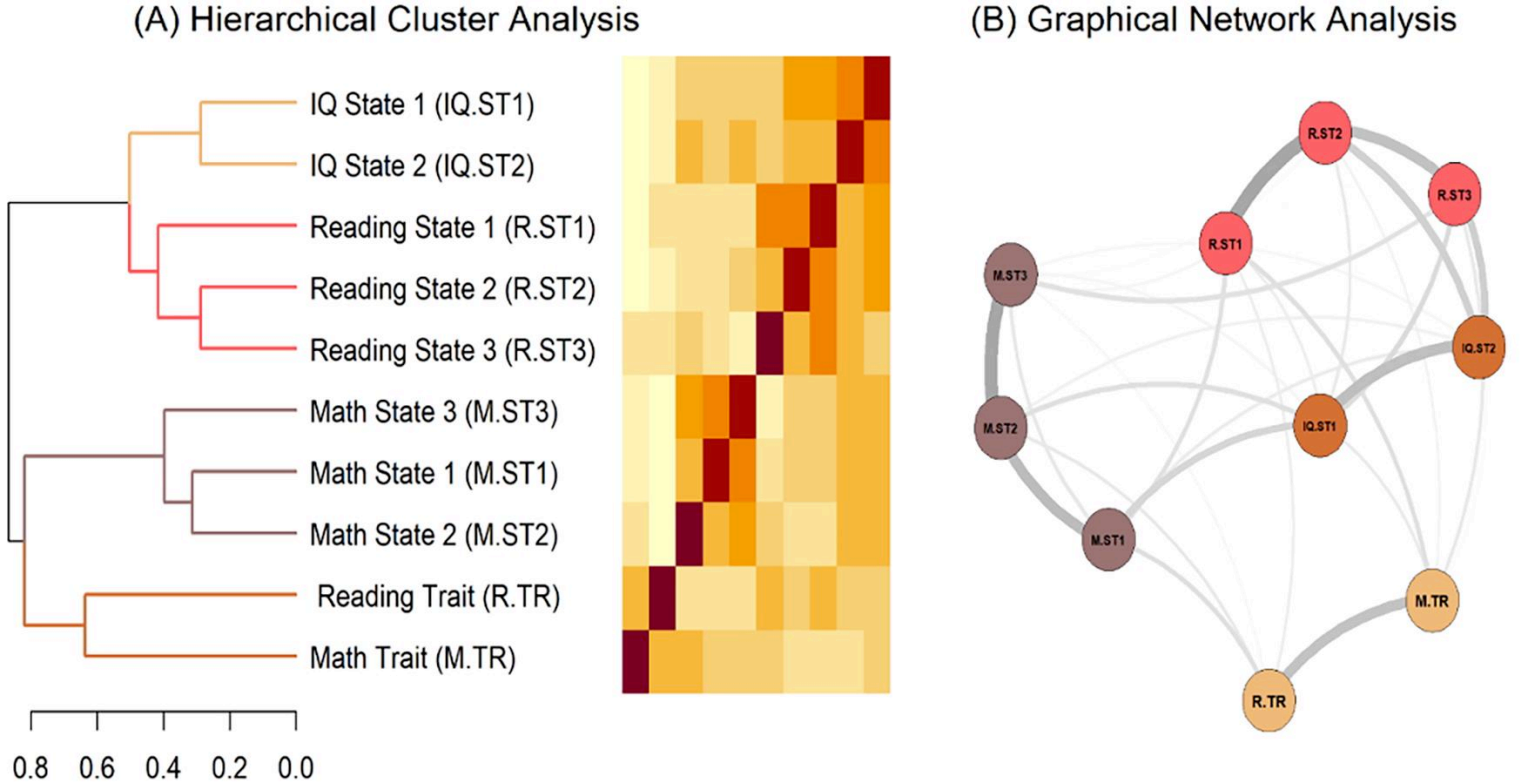
Structural analyses of anxiety scales. **(A)** Dendrogram of the hierarchical cluster analysis with corresponding distance matrix (based on Pearson correlations) and **(B)** Graphical Network Analysis (GGM with EBICglasso operator).

Next, we tested how the various anxiety scales administered in this study are clustered. Figure 4 displays (A) the dendrogram of the hierarchical cluster analysis and the corresponding distance matrix based on Pearson correlations and (B) the results of the network analysis. Although all anxiety scales are somehow related, it is to note that the scales are clustered according to their respective domains, this means, according to whether they are expected to measure IQ test, reading, or math anxiety—despite all of these scales use identical item formats. Both trait scales also form a cluster, but these measures differ both in form (item wordings) and measurement intention (stable components) from all state scales.

#### 3.1.2 State characteristics of reading and relations to test performance (RQ2)

**Figure 5:**
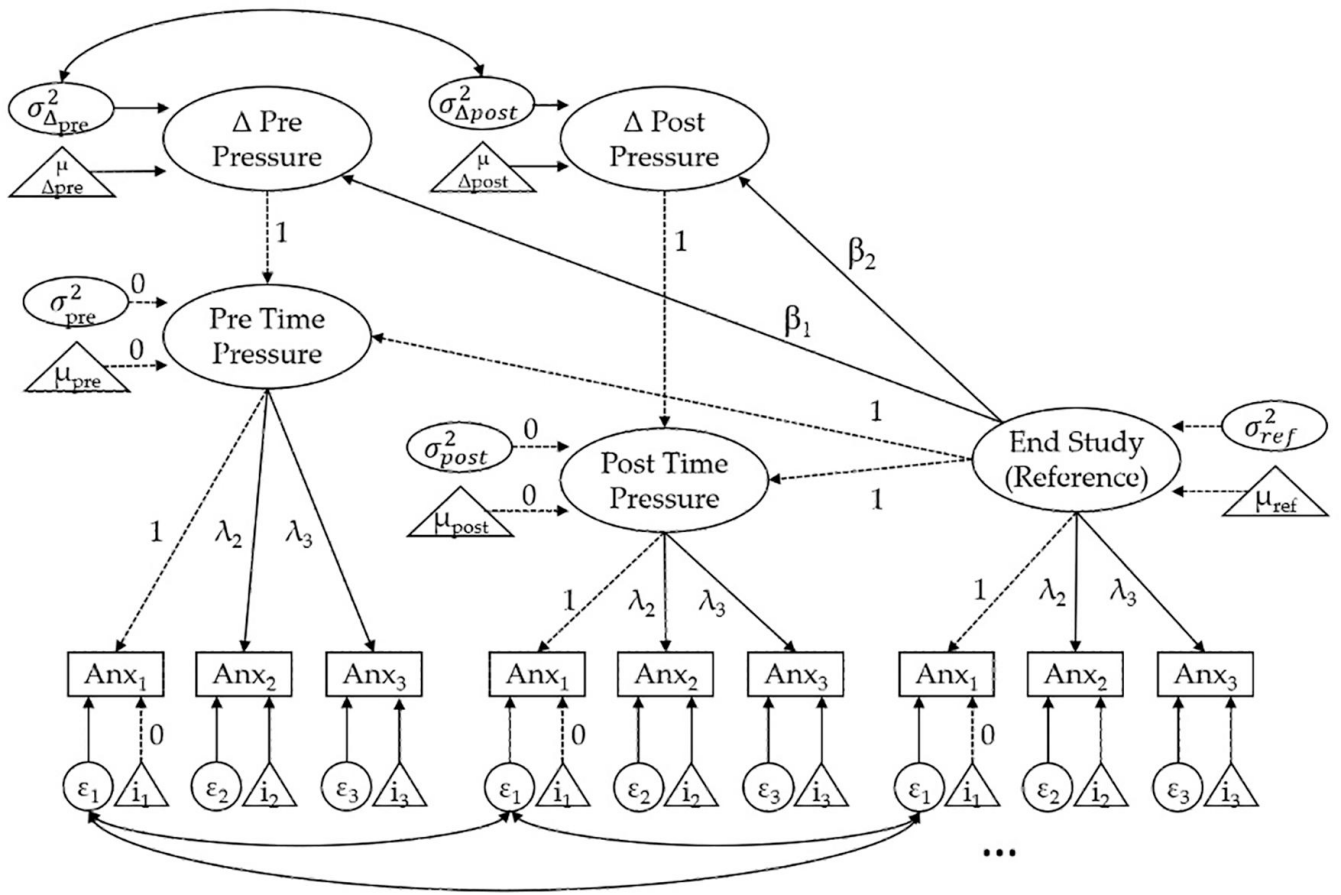
Latent change score (LCS) model of state anxiety.

The LCS model depicted in Figure 5 was used to simultaneously test level and changes of state anxiety. Further, trait anxiety and objective performance correlates in the respective domains have been added (not shown in Figure 5) to test their relations. Residuals of corresponding indicator variables were allowed to be correlated across measurement points.

Consider first the reading domain. The LCS model with correlates revealed good measurement models (see Supplement S4) and yielded a close fit to data (*Χ*^2^(67) = 76.39, *p* = .20; RMSEA = .02 [95% CI:.00;.04]; SRMR = .05; CFI = .99; TLI = .99). The mean anxiety level (factor mean) of the reference condition (state 3) was estimated to be *μ*_ref_ = 1.094 (SE = 0.059; *p* < .001) in the metric of the reference indicator. The relative increases in factor means to the conditions surrounding the time-pressure reading test were μ_Δpre_ = 0.712 (SE = 0.093; *p* < .001) and μ_Δpost_ = 0.355 (SE = 0.077; *p* < .001) for state 1 and 2, respectively. The relations of the factors are displayed in Table 2 above the diagonal. The relative change in variance surrounding the time-pressure test ($\sigma_{\Delta \mathrm{pre}}^{2}$ and $\sigma_{\Delta \mathrm{post}}^{2}$) were negatively related (*β*_1_ = −.55, *p* < .001; *β*_2_ = −.40, *p* < .001) with the reference condition ($\sigma_{\mathrm{ref}}^{2}$), suggesting, in line with the trajectories in Figure 3, that the increase in state anxiety in participants with generally increased levels of anxiety were not as pronounced as those who experienced lower levels of anxiety in the reference condition. However, both change factors (pre and post-test) were positively related (*r* = .73, *p* < .001), indicating that the magnitude of changes is comparable for both states surrounding the time-pressure test. As could be expected, trait reading anxiety was consistently related with state anxiety in the reference condition (state 3: *r* = .41, *p* < .001) which was assumed to capture baseline levels of anxiety. A weaker relation of trait anxiety was observed with the change-score in the pre-test condition (state 1: *r* = .35, *p* < .001), whereas there was no relation with the change score in the post-test condition (state 2: *r* = .07, *p* = .44). Both reading performances under time-pressure and without pressure were only moderately related (*r* = .19, *p* < .001), suggesting that the time-pressure manipulation changes the nature of the test considerably. Performance under time pressure was moderately inversely related with trait anxiety (*r* = −.17, *p* = .02) and state anxiety in the reference condition (*r* = −.24, *p* < .01) as well the relative change in the pre-test (*r* = −.22, *p* < .01). By contrast, performance in the test without time-pressure was neither related with trait anxiety (*r* = −.08, *p* = .25) nor state anxiety in the reference condition (*r* = .00, *p* = .97). By contrast, moderately positive relations with task performance were observed for the relative changes in both change scores (change at pre-test: *r* = .20, *p*.02; change at post-test: *r* = .24, *p* < .01).

**Table 2:** Latent relations of LCS variables with correlates.

	Factors of LCS model			Correlates		
	Reference	*Δ* Pre	*Δ* Post	Trait	Perf TP	Perf NP
Reference	–	-.55***	-.40***	.41***	-.24***	.00
Δ Pre	−.52***	–	.73***	.35***	−.22**	.20*
Δ Post	−.26***	.53***	–	.07	-.01	.24**
Trait	.37***	.27***	.17*	–	−.17*	−.08
Perf TP	−.19**	−.04	−.04	−.23***	–	.19***
Perf NP	−17**	−.10	.01	−.28***	.63***	–

Standardized latent relations of LCS factors with correlates for reading and math domains in the upper and lower triangle, respectively. LCS model for anxiety: Reference = anxiety at end of the study, Δ Pre and Δ Post = changes relative to reference in the pre- and post-time pressure conditions, respectively. Correlates: Trait = trait anxiety, Perf TP and Perf NP = Performance in a time-pressure and in a non-time pressure condition in a corresponding ability test.

* *p* < .05.

** *p* < .01.

*** *p* < .001.

Consider next the math domain. An analogous model displayed again good measurement models (see Supplement S4) and revealed a close fit to the data (*Χ*^2^(67) = 89.10, *p* = .04; RMSEA = .03 [95% CI:.01;.05]; SRMR = .04; CFI = .99; TLI = .98). The anxiety level in the reference condition was estimated to be μ_ref_ = 1.034 (SE = 0.064; *p* < .001). The relative increases to the conditions surrounding the time-pressure test were μ_Δpre_ = 0.667 (SE = 0.079; *p* < .001) and μ_Δpost_ = 0.497 (SE = 0.080; *p* < .001), respectively. The relations of the factors are displayed in Table 2 below the diagonal. Again, both change scores were negatively related with anxiety in the reference condition (*β*_1_ = −.52, *p* < .001; *β*_2_ = −.26, *p* < .001), but were mutually positively correlated (*r* = .53, *p* < .001). Also in the math domain, trait anxiety was strongest related (in relative terms) with state anxiety in the reference condition (*r* = .37, *p* < .001), less so with increases in pre-test (*r* = .27, *p* < .001) and even weaker with the increase in post-test (*r* = .17, *p* < .06). Math performance was considerably more strongly related in both time-pressure and non-pressure conditions (*r* = .63, *p* < .001). Both trait anxiety and state anxiety in the reference condition were inversely related with math performance in both test conditions (trait anxiety: *r* = −.23, *p* < .001; *r* = −.28, *p* < .001; state anxiety: *r* = −.19, *p* < .01; *r* = −.17, *p* < .01, for the time pressure and non-pressure tests, respectively). By contrast, none of the change scores was substantially related with test performance (all |*r*| ≤ .10, all *p* ≥ .15).

Given the moderate gender effects previously observed in some of the scale means, we doublechecked the robustness of the SEM analyses by removing (residualizing) gender from all observed variables. This resulted in only minor numeric differences, but did not alter the results.

#### 3.1.3 Testing moderation effects of test anxiety (RQ3)

We tested if specific forms of anxiety affect and qualify the effects of general cognitive ability on performance in each of the four performance tests as dependent variables (i.e., reading and math tests, each applied under time pressure and without pressure). Predictors in each regression were: general cognitive ability (fluid intelligence), one of the anxiety measures, and the interaction of both. Results are summarized in Supplement S5. Across all regression models, general cognitive ability (fluid intelligence) was the strongest predictor of test performance (.37 ≤ *β* ≤ .52). Only predictions of performance in the non-pressure reading test were considerably more moderate (.21 ≤ *β* ≤ .22). With the exception of regression models predicting performance in the reading text without time pressure, the alternative measures of anxiety were moderately inversely related with test performance, somewhat less consistent across regression models (−.07 ≤ *β* ≤ −.22). Further, there were no meaningful interaction effects (all very close to zero, and all statistically non-significant). Again, reading without pressure deviated from this pattern, with positive regression coefficients for state 1 and 2 (*β* = .12; *β* = .11), but virtually zero effects for state 3 and for trait anxiety. However, there was a moderate negative moderation effect (*β* = −.14) in state 1 and 2, indicating that the otherwise positive effects of ability are attenuated at high levels of state anxiety in the non-pressure reading test.

## 4 Discussion

Given the limited research on the role of anxiety in reading contexts (9), the present study sought to advance the conceptualization of RA in school-aged children by embedding it within the state-trait anxiety framework and by directly comparing it with MA under conditions with and without time pressure. Adopting a state-trait-model may be appropriate for RA, as the more extensive research on MA has revealed discrepancies between trait-based self-report questionnaires and real-time, state-based assessments of anxiety (10, 48, 49). These inconsistencies in the MA literature have been attributed, in part, to actual state-trait dynamics of perceived anxiety as well as differences in questionnaires employed for their respective assessment (19, 20, 44). To date, the few existing studies on RA have largely relied on self-report instruments originally developed for MA. These instruments do not clearly distinguish between state and trait components of anxiety, which may limit a precise conceptualization of RA.

### 4.1 Psychometrics and structure of anxiety measures (RQ 1)

In light of these challenges, the first research question addressed the psychometric characteristics and latent structure of state and trait measures of RA among other anxiety measures, including math and general IQ test anxiety. All anxiety measures exhibited satisfactory psychometric properties, with high internal consistency and strong factor loadings of all items. The range of bias-corrected bootstrap intervals suggested that un-corrected estimates may be slightly elevated because of the right-skewed distributions, as is frequently observed in clinical data. However, even the conservative estimates proved to be satisfactory for the employed short scales. Confirmatory factor analyses further supported the uni-dimensionality of the trait anxiety measures. For the state anxiety measures, model fit was improved in some cases by allowing correlated error variances between pairs of semantically similar items. Taken together, the results indicate that all scales were appropriate for the intended analyses. Building on the assumption that anxiety is best characterized as a hierarchical construct, we additionally examined the clustering of the administered anxiety measures. The resulting hierarchical clustering closely mirrored the intended measurement intentions of the scales. State measures of RA, MA and IQ anxiety formed separable clusters, despite all instruments employed identical item formats. This pattern was corroborated in the network analysis and provides evidence for the discriminant validity of the measures, even though all scales were positively correlated in principle. Furthermore, trait anxiety measures formed a separate cluster, supporting the state-trait distinction across both domains. In analogy to MA, these results provide converging evidence that RA can be coherently framed within a trait-state conceptualization. Similar to the adapted version of the MAQ (20, 70), which assesses anxiety related to failure in math, the newly developed trait-Reading-Anxiety-Questionnaire (trait-RAQ) targets trait anxiety dispositions related to failure in reading, thus extending the trait-state framework to the reading domain.

### 4.2 State characteristics of RA and MA and correlates (RQ 2)

#### 4.2.1 State characteristics of RA and MA

To date, empirical evidence on the effects of time pressure on state anxiety in children's academic performance is limited. There are some indications that test situations with time pressure are associated with elevated levels of MA and this might affect the selection of problem-solving strategies. Children might tend to apply less accurate strategies under time pressure (56, 57). To the best of our knowledge, no study has systematically examined the interplay between experimentally manipulated time pressure and children's state anxiety in the domain of reading. Therefore, the second research question examined differences in children's state anxiety in test situations with and without time pressure, as well as in comparison to a non-threatening reference condition (baseline state anxiety).

The data indicate that state anxiety in both domains is systematically influenced by time pressure, while at the same time showing a high degree of rank stability. Overall, state anxiety levels increased significantly in the measurements conducted before and after test conditions with time pressure compared to the non-threatening reference condition. However, these increases were less pronounced among participants who already exhibited elevated state anxiety levels in the reference condition, suggesting reduced situational reactivity among children with generally elevated state anxiety. Interestingly, RA revealed no clear gender differences in the present study. Likewise, there were no consistent differences in objectively assessed reading performance. By contrast, MA was found to be increased in girls relative to boys, and accompanied by inverse effect in math performance. While these findings in the math domain are consistent with prior research, the absence of comparable gender differences in the reading domain represents a novel contribution. Further research is needed to replicate and extend these findings. The LCS model demonstrated a close fit to data for both reading and math domains, indicating that the assumed distinction between baseline state anxiety and situational state anxiety changes provides an adequate account of the observed data. As expected, trait anxiety was most strongly related with state anxiety in the reference condition, supporting the notion that this measurement situation can serve as a baseline estimate. Weaker associations were observed between trait anxiety and anticipatory state anxiety changes prior to the test, whereas little to no association was found with retrospectively reported state anxiety changes following the test.

In both domains, state anxiety levels in the reference condition were found to be negatively associated with relative anxiety increases in the reports prior and post time-pressure testing. This negative correlation does not necessarily imply a reduction of anxiety under time pressure but rather reflects a reduced reactivity among participants with generally elevated state anxiety levels. Conversely, children with lower baseline state anxiety showed stronger situational modulation of their anxiety responses. In sum, time pressure elicits situational increases in children's state anxiety, but the magnitude and functional significance of these increases depend on baseline state anxiety levels.

#### 4.2.2 Relations with academic performance

Since the beginning of research on academic anxieties, the relation of anxiety and academic performance has been a central concern (53, 91). For some time, research on the relationship between MA and math performance in younger children yielded discrepant findings. Analyses of assessment approaches suggested that these inconsistencies could be partly explained by differences between the questionnaires (20, 44). Consistent with this interpretation, meta-analyses indicate that the type of MA questionnaire moderates the strength of the association between MA and math fluency (12–14). It therefore appears likely that real-time assessments of MA (state anxiety) or self-reports capturing children's affective experiences in specific math situations show a closer association with math fluency than trait-based instruments that primarily assess a generalized fear of failure in math (20, 44). State anxiety measures reflect affective experiences during task engagement and are thus more proximal to the performance process, whereas trait measures capture more stable dispositions or stable self-concepts that may be less directly related to situational cognitive functioning during task processing (46).

Regarding the relationships of anxiety and performance, the present findings reveal a domain-specific pattern, indicating that the effects of time pressure and anxiety differ between reading and math. Time pressure may interact with domain-specific task demands and anxiety processes in distinct ways.

In the reading domain, performance in the time-pressure and non-pressure conditions were only weakly related, suggesting that reading performance was considerably affected by the time-pressure manipulation. This interpretation is supported by the observation that reading performance was differentially associated with intelligence, with markedly stronger relations in the time-pressure condition than in the non-pressure condition. Moreover, the LCS analyses show a differentiated pattern of associations between anxiety and reading performance. Specifically, reading performance under time pressure was negatively related to trait anxiety, baseline state RA, and anticipatory increases in state RA, whereas retrospective RA changes showed no meaningful association. In contrast, reading performance in the non-pressure condition was not related to trait RA or baseline state RA. Instead, only the anxiety change scores were positively associated with performance. One possible interpretation is that moderate situational increases in state RA may facilitate performance under otherwise low-demand, non-threatening conditions, consistent with notions of optimal arousal or facilitative anxiety in relatively relaxed testing contexts.

An alternative explanation could be that the steepest state RA increases were observed among children with overall low state RA levels, who may also have been less anxious during the non-pressure test. However, this account appears insufficient, as neither trait RA nor baseline state RA was inversely related to performance in the non-pressure condition. Moreover, this explanation does not readily account for the pronounced differences in anxiety-performance relations observed between the time-pressure and non-pressure reading conditions.

In the math domain, performance under time-pressure and non-pressure conditions was substantially more strongly related, suggesting that the pressure manipulation had a weaker impact on math performance than in reading. Consistent with this interpretation, the pattern of associations between anxiety and performance was largely comparable across both math test conditions. In both cases, performance was inversely related to trait MA and baseline state MA, whereas situational state MA changes showed no meaningful relationship with performance. This pattern suggests that trait anxiety effects were more pronounced in math, both with respect to test performance as well as with respect to anxiety.

Importantly, these findings do not preclude the existence of state anxiety effects in math. Prior research has demonstrated such effects, and it has been argued that different forms of MA may differentially affect performance depending on specific task demands (10, 48, 92). There are indications that distinct subtypes of MA exist, ranging from potentially performance-facilitating effects to the more commonly assumed performance-inhibiting effects (38, 93, 94). In addition, the time-pressure and non-pressure math tests employed in the present study differed in format, and their degree of similarity may have influenced the observed relations. Finally, the math assessments were administered in the latter part of an extensive test battery, raising the possibility that state MA responses may have been attenuated due to habituation or fatigue compared to the earlier reading assessments.

In summary, the findings show that time pressure induces situational increases in state anxiety, but that the magnitude and functional relevance of these increases depend strongly on children's baseline state anxiety levels. While the overall structure of state anxiety responses was largely comparable across reading and math, the links between anxiety and performance differed markedly between domains. Specifically, anxiety-performance associations in reading emerged primarily under time-pressure conditions, highlighting the role of anticipatory state anxiety in reading performance when tasks are time-constrained.

#### 4.2.3 Testing moderation effects of test anxiety (RQ3)

For a long time, research has predominantly examined the relationship between anxiety and academic performance by focusing on general associations between these factors. Beyond avoidance behavior, performance decrements under anxiety have been explained by interference within the attentional control system (30). As a result, the interplay between affective and cognitive factors has received increasing attention in MA research in recent years (52). To our knowledge, comparable research approaches have not yet been systematically applied to RA.

In contrast to the extensive literature on the interaction of MA with specific cognitive abilities such as WM or EF, considerably less attention has been paid to the role of intelligence. While some studies have reported no association between intelligence and trait-like dispositions of MA (53, 54), one study identified a small but significant correlation between intelligence and state MA (10). Thus, despite previous research ruling likely that anxiety and cognitive ability may interact, the question to what extend anxiety and general cognitive ability jointly predict performance in reading and math domains remains largely unexplored. Therefore, the last research question examined whether domain-specific anxiety moderates the relationship between intelligence and test performance, thereby addressing how affective and cognitive factors jointly influence academic performance.

Based on the present data, the role of intelligence and anxiety can be differentiated more clearly. Across most test conditions, intelligence emerged as the strongest positive predictor of performance, whereas domain-specific anxiety showed moderate negative associations. Importantly, the lack of interaction effects suggests that intelligence and anxiety contributed to performance outcomes largely independently of each other. This pattern aligns with previous findings reporting little to no direct associations between intelligence and anxiety that have been used as predictor variables here. This suggests that higher cognitive ability does not generally serve as a buffer against adverse effects of anxiety, nor does anxiety systematically diminish the benefits of higher ability.

An exception to this pattern was observed in the non-pressure reading condition. Here, intelligence was a weaker predictor of performance, as the task had generally less of a cognitive demand. By contrast, anxiety was positively associated with test performance, suggesting a possible facilitative effect of anxiety in an easy task context. Notably, only in this condition did anxiety moderate the ability–performance relationship, such that the positive effect of cognitive ability was attenuated at higher levels of state anxiety. This finding implies that under specific task conditions, anxiety may interfere with the effective deployment of cognitive ability.

Taken together, the data suggest that intelligence and anxiety are largely independent predictors of performance, with limited evidence that children with different levels of cognitive ability are systematically more or less affected by domain-specific anxiety. However, the exceptional observation of the moderation effect suggests that under certain conditions, anxiety may alter the effects of cognitive ability on test performance. Given that this pattern emerged in only one test condition in this study, conclusions regarding differential susceptibility based on intelligence should be drawn cautiously and warrant replication in future studies.

## 5 Limitations

Several limitations of the present study should be acknowledged. Firstly, different instruments were used to assess performance under time pressure and without time pressure in both reading and math. This was done to reduce the impact of test repetition and training effects that would have otherwise occurred when using identical performance tests. However, using different tests restricts the comparability of performance across conditions and may have contributed to differential patterns of associations with anxiety and intelligence, especially in the reading domain. In addition, reading performance was assessed exclusively using reading comprehension tests, without including an oral or read-aloud situation. Reading tasks involving oral performance or public evaluation may elicit higher levels of state anxiety and different performance effects, which limits the generalizability of the present findings to such contexts. Secondly, although the overall sample size was adequate, some analyses may have been underpowered to detect small interaction effects, potentially limiting sensitivity to subtle moderating influences. Thirdly, the cross-sectional design precludes causal conclusions regarding the directionality of the relationships between anxiety, intelligence and performance. Longitudinal studies are needed to clarify developmental trajectories, and reciprocal influences. Fourth, in the one-factorial CFAs used to test the measurement models for each scale, we used MLR estimation with simultaneous FIML imputation of missing values. Given that indicators were used that permitted only five-step graded responses, a robust estimator fitted to ordered responses could have been used instead at the cost of excluding incomplete cases or requiring imputations of missing values prior to the analyses. Hence, we double checked results for all scales using the robust DWLS estimator fitted to complete cases (260 ≤ *N* < 283) which confirmed all findings with slightly stronger loadings of the items (minimum *λ* = .45) and somewhat improved model fits. Fifth, while construct saturation was generally satisfactorily high in all scales, few of the state items displayed weak loadings by conventional standards. However, this occurred only rarely and inconsistently across the items of the state scales (see Supplement S1). Therefore, we decided to keep all items so that measurement models comprised identical items in all state scales. This also avoided a bias driven by item content in the subsequent cluster and network analyses. Sixth, we permitted residual covariances for two pairs of synonymous items for theoretical reasons and to improve model fit (confirmed by formal model comparisons and bootstrapping). However, as model improvements always bear the risk of overfitting, replications using independent samples are recommended to consolidate the structure. Seventh, relatedly, the newly developed trait RAQ was tested here for the first time, and more research is required to consolidate its psychometric properties. This would need to comprise evidence on test-retest reliability, tests on content validity, and an expansion of the tests of construct validity including other established scales. Eighth the fixed order of the study design facilitates potential sequence effects (e.g., the carryover of emotional states, habituation, and exhaustion) which could have contributed to the differences found in the reading and in the math domains. Further limitations concern the interpretation of the LCS. Although LCS models address some shortcomings of traditional difference scores, the strong negative relation between change scores and the reference condition, as well as the high correlations among change scores, may partly reflect effects of the reference measurement. Moreover, the reference condition was assessed at the end of the testing session. It is possible that anxious children may have had difficulties in downregulating their anxiety, which could have resulted in an attenuating of the estimated change effects. Future studies should assess baseline state anxiety prior to task performance, ideally with greater temporal separation and multiple baseline measurements. Finally, state anxiety was assessed using real-time assessments but relied on self-ratings rather than physiological indicators. The incorporating of multimethod approaches, such as behavioral or physiological measures, would strengthen future research.

## 6 Conclusions

The present study aimed to advance research on RA by embedding it within a state-trait framework and by directly comparing it with MA under conditions with and without time pressure. The findings demonstrate that RA, similar to MA, can be conceptualized as a hierarchical construct comprising distinct yet related state and trait components. This conceptualization is supported by robust psychometric properties and clear evidence of domain specificity.

Across both domains, time pressure reliably elicited increases in state anxiety, while individual differences in baseline state anxiety substantially shaped the magnitude of these situational responses. Children with lower baseline state anxiety showed stronger anxiety increases under time pressure, whereas children with generally elevated state anxiety displayed reduced situational reactivity. These findings underline the importance of considering baseline state anxiety when interpreting situational state anxiety responses in academic contexts.

Importantly, the associations between anxiety and performance differed between reading and math. In reading, anxiety-performance relations emerged primarily under time-pressure conditions, with higher trait anxiety, elevated baseline state anxiety, and anticipatory RA increases predicting poorer performance. By contrast, moderate situational increases in anxiety appeared to be facilitative in non-threatening reading contexts. In math, performance was consistently related to trait and baseline state MA across test conditions, indicating a stronger influence of stable anxiety dispositions in this domain.

To conclude, the findings highlight the domain-specific nature of anxiety effects in reading and math and emphasize the value of a state–trait perspective for understanding how anxiety influences children's academic performance. By distinguishing baseline state anxiety levels, situational state changes, and more stable trait dispositions, the present study contributes to a more differentiated framework for understanding anxiety-performance relations in school-aged children and offers a conceptual basis for future research that explicitly accounts for variability in anxiety processes across domains and task contexts.

## Data Availability

The raw data supporting the conclusions of this article will be made available by the authors, without undue reservation.
