# Supplementary material for: Reading and math anxiety in children: differential roles of state and trait components in academic performance, and the moderating effects of intelligence and time pressure
Source: Front Child Adolesc Psychiatry. 2026 May 8;5:1778068. doi: 10.3389/frcha.2026.1778068 (PMC13199927; doi:10.3389/frcha.2026.1778068)
Supplement: Supplementary file 1 [file Supplementaryfile1.pdf]

Supplement S1.

Fit of CFA measurement models

#	Scale	X^2 (*df*), *p*	RMSEA [95% CI]	SRMR	CFI	TLI	Loadings *M* [Range]
1	Reading Anxiety Trait	27.45(14), .02	.06 [.03;.08]	.03	.97	.96	.72 [.63;.78]
2	Reading Anxiety State 1	13.67(12), .32	.02 [.00;.06]	.03	1.00	.99	.55 [.33;.80]
3	Reading Anxiety State 2	41.78(12), .00	.09 [.07;.12]	.06	.93	.88	.58 [.38;.83]
4	Reading Anxiety State 3	22.11(12), .04	.05 [.02;.08]	.04	.98	.96	.64 [.46;.86]
5	Math Anxiety Trait	22.03(14), .08	.05 [.01;.07]	.03	.99	.98	.79 [.70;.85]
6	Math Anxiety State 1	14.36(12), .28	.03 [.00;.06]	.03	.99	.99	.64 [.38;.80]
7	Math Anxiety State 2	14.53(12), .27	.03 [.00;.06]	.03	.99	.99	.70 [.57;.80]
8	Math Anxiety State 3	18.04(12), .11	.04 [.00;.07]	.04	.99	.98	.71 [.49;.91]
9	IQ Test Anxiety State 1	24.81(12), .02	.06 [.03;.09]	.04	.98	.96	.69 [.46;.86]
10	IQ Test Anxiety State 2	28.79(12), .00	.07 [.04;.10]	.05	.97	.95	.67 [.49;.86]

Note. All scales comprised of 7 items. Two residual correlations were allowed for two highly synonymous item pairs in all state scales ($\theta_{\varepsilon 1,2}$; $\theta_{\varepsilon 5,6}$), see Supplement S2 for details.
