# Supplementary material for: Reading and math anxiety in children: differential roles of state and trait components in academic performance, and the moderating effects of intelligence and time pressure
Source: Front Child Adolesc Psychiatry. 2026 May 8;5:1778068. doi: 10.3389/frcha.2026.1778068 (PMC13199927; doi:10.3389/frcha.2026.1778068)
Supplement: Supplementary file 2 [file Supplementaryfile2.pdf]

Supplement S2.

Test of Residual Correlations

#	Scale	Residual Correlations		Separate Test of $\theta_{\varepsilon 1,2}$			Separate Test of $\theta_{\varepsilon 5,6}$			Simultaneous Test of $\theta_{\varepsilon 1,2}$ and $\theta_{\varepsilon 5,6}$		
		$\theta_{\varepsilon 1,2}$ [95% BI]	$\theta_{\varepsilon 5,6}$ [95% BI]	$X^2(df), p$	ΔAIC	ΔBIC	$X^2(df), p$	ΔAIC	ΔBIC	$X^2(df), p$	ΔAIC	ΔBIC
2	Reading Anxiety State 1	.38 [.25;.48]	.15 [.02;.28]	39.12(1), <.001	-33.59	-29.94	4.39(1), .036	-2.75	0.89	41.65(2), <.001	-37.30	-30.00
3	Reading Anxiety State 2	.40 [.27;.51]	.37 [.24;.49]	67.02(1), <.001	-35.58	-31.93	28.52(1), <.001	-31.74	-28.09	75.88(2), <.001	-71.63	-64.34
4	Reading Anxiety State 3	.43 [.28;.54]	.46 [.32;.59]	31.70(1), <.001	-42.89	-39.24	28.46(1), <.001	-52.89	-49.24	66.25(2), <.001	-98.77	-91.48
6	Math Anxiety State 1	.27 [.13;.41]	.39 [.25;.52]	9.10(1), .003	-13.38	-9.75	24.32(1), <.001	-39.64	-36.01	33.44(2), <.001	-53.06	-45.79
7	Math Anxiety State 2	.32 [.16;.46]	.42 [.25;.57]	13.99(1), <.001	-20.11	-16.49	22.62(1), <.001	-45.22	-41.59	37.92(2), <.001	-64.66	-57.41
8	Math Anxiety State 3	.51 [.39;.62]	.51 [.36;.64]	32.23(1), <.001	-63.27	-59.66	23.77(1), <.001	-68.47	-64.86	57.14(2), <.001	-136.46	-129.25
9	IQ Test Anxiety State 1	.42 [.29;.53]	.38 [.21;.53]	33.72(1), <.001	-43.76	-40.10	17.63(1), <.001	-29.35	-25.69	51.11(2), <.001	-76.71	-69.39
10	IQ Test Anxiety State 2	.49 [.36;.61]	.29 [.13;.43]	31.35(1), <.001	-62.76	-59.10	10.14(1), .001	-16.93	-13.27	45.70(2), <.001	-83.03	-75.72

Note. Residual correlations $\theta_{\varepsilon 1,2}$ and $\theta_{\varepsilon 5,6}$, are reported fully standardized [with 95% bootstrap intervals, based on 1000 resamples]. Both residual correlations were first tested separately, then simultaneously as compared with a model without residual correlations. $X^2(df), p$ denotes the reduction in chi-squared with corresponding p-value (large and significant values indicate better fit of the model with residual correlations). ΔAIC and ΔBIC denote the change in respective comparison indices (with negative values indicating better fit of the model with residual correlations).
