# Supplementary material for: Reading and math anxiety in children: differential roles of state and trait components in academic performance, and the moderating effects of intelligence and time pressure
Source: Front Child Adolesc Psychiatry. 2026 May 8;5:1778068. doi: 10.3389/frcha.2026.1778068 (PMC13199927; doi:10.3389/frcha.2026.1778068)
Supplement: Supplementary file 3 [file Supplementaryfile3.pdf]

Supplement S3.

Tests of gender differences in all scales

#	Scale	Female (*M*, *SD*)	Male (*M*, *SD*)	*t*(*df*), *p*	Cohen's *d* [95% CI]
1	Reading Anxiety Trait	11.015 (6.253)	9.598 (6.625)	1.801(265), .073	0.220 [-0.021;0.461]
2	Reading Anxiety State 1	6.898 (4.619)	6.029 (4.798)	1.549(277), .123	0.185 [-0.050;0.419]
3	Reading Anxiety State 2	5.870 (4.755)	5.650 (5.113)	0.375(276), .708	0.045 [-0.190;0.279]
4	Reading Anxiety State 3	5.270 (4.865)	5.106 (5.585)	0.257(260), .797	0.031 [-0.207;0.270]
5	Math Anxiety Trait	10.752 (7.078)	8.913 (8.080)	1.948(250), .053	0.242 [-0.003;0.488]
6	Math Anxiety State 1	7.378 (5.250)	5.733 (5.352)	2.584(274), .010*	0.310 [0.073;0.548]
7	Math Anxiety State 2	7.636 (6.021)	5.978 (6.076)	2.285(275), .023*	0.274 [0.037;0.512]
8	Math Anxiety State 3	5.674 (5.716)	4.320 (5.602)	1.950(263), .052	0.239 [-0.003;0.482]
9	IQ Test Anxiety State 1	6.958 (5.341)	6.820 (6.087)	0.203(274), .839	0.024 [-0.210;0.258]
10	IQ Test Anxiety State 2	7.174 (5.794)	6.714 (6.254)	0.633(268), .528	0.076 [-0.161;0.313]
11	Reading Perf Time Press	-0.073 (1.057)	0.077 (0.933)	-1.264(281), .207	-0.150 [-0.384;0.084]
12	Reading Perf No Press	0.073 (0.998)	-0.078 (1.000)	1.251(274), .212	0.150 [-0.087;0.387]
13	Math Perf Time Press	-0.155 (0.965)	0.165 (1.014)	-2.691(271), .008**	-0.324 [-0.562;-0.086]
14	Math Perf No Press	-0.205 (1.033)	0.216 (0.919)	-3.555(268), <.001***	-0.431 [-0.673;-0.189]

Note. The reading and math performance scores were averaged from two standardized raw indicators, see text for further information. Cohen's *d* with Hedges correction; positive numbers indicate larger values of female relative to male participants.

*p < .05.
**p < .01.
***p < .001.
