# Supplementary material for: Reading and math anxiety in children: differential roles of state and trait components in academic performance, and the moderating effects of intelligence and time pressure
Source: Front Child Adolesc Psychiatry. 2026 May 8;5:1778068. doi: 10.3389/frcha.2026.1778068 (PMC13199927; doi:10.3389/frcha.2026.1778068)
Supplement: Supplementary file 4 [file Supplementaryfile4.pdf]

Supplement S4.

Measurement models of the LCS analyses

Model	State 1 (Pre Time Pressure)			State 2 (Post Time Pressure)			State 3 (Reference)			Trait		
	λ_{11}	λ_{21}	λ_{31}	λ_{12}	λ_{22}	λ_{32}	λ_{13}	λ_{23}	λ_{33}	λ_{14}	λ_{24}	λ_{34}
Reading	.69	.65	.60	.71	.67	.64	.73	.73	.65	.83	.89	.85
Math	.75	.79	.63	.80	.83	.69	.78	.84	.66	.92	.92	.86

Note. λ coefficients are completely standardized loadings. In the state measurement models, all corresponding parameters were constrained to be equal across states prior to standardization. For the trait measurement models, they were estimated freely.
