# Supplementary material for: Reading and math anxiety in children: differential roles of state and trait components in academic performance, and the moderating effects of intelligence and time pressure
Source: Front Child Adolesc Psychiatry. 2026 May 8;5:1778068. doi: 10.3389/frcha.2026.1778068 (PMC13199927; doi:10.3389/frcha.2026.1778068)
Supplement: Supplementary file 5 [file Supplementaryfile5.pdf]

Supplement S5.

Tests of the moderation analyses

	Reading						Mathematics					
	Time Pressure			Non-Pressure			Time Pressure			Non-Pressure		
Model	*β*	*p*	R^2	*β*	*p*	R^2	*β*	*p*	R^2	*β*	*p*	R^2
Intelligence	.37	<.01***		.22	<.01***		.51	<.01***		.45	<.01***	
Anxiety State 1	-.22	<.01***		.12	.04*		-.07	.16		-.11	.05*	
Interaction	-.01	.90	.21	-.14	.02*	.08	-.01	.86	.28	-.04	.51	.23
Intelligence	.39	<.01***		.23	<.01***		.52	<.01***		.45	<.01***	
Anxiety State 2	-.14	.01*		.11	.07		-.11	.03*		-.09	.12	
Interaction	-.05	.35	.19	-.14	.02*	.08	.07	.16	.29	-.09	.12	.23
Intelligence	.37	<.01***		.21	<.01***		.52	<.01***		.45	<.01***	
Anxiety State 3	-.17	<.01**		.01	.87		-.14	<.01**		-.12	.03*	
Interaction	-.02	.67	.18	-.11	.07	.06	-.02	.69	.30	-.09	.11	.24
Intelligence	.38	<.01***		.22	<.01***		.49	<.01***		.43	<.01***	
Anxiety Trait	-.11	.05*		-.02	.72		-.14	<.01**		-.19	<.01***	
Interaction	.02	.70	.17	-.01	.82	.05	.05	.33	.29	.02	.65	.25

Note. Four separate moderation analyses were conducted for each performance test as a dependent variable (as indicated by the column headers). Each regression model (separated by horizontal lines) included as predictors: intelligence, one of the anxiety scales in the respective domain (as specified in the first column) and their interaction. For each predictor, *β* = standardized regression coefficient and *p* = corresponding *p*-value are given. R^2 = The variance accounted for in each model.

*p < .05.

**p < .01.

***p < .001.
