# Supplementary material for: Reading and math anxiety in children: differential roles of state and trait components in academic performance, and the moderating effects of intelligence and time pressure
Source: Front Child Adolesc Psychiatry. 2026 May 8;5:1778068. doi: 10.3389/frcha.2026.1778068 (PMC13199927; doi:10.3389/frcha.2026.1778068)
Supplement: Supplementary file 6 [file Supplementaryfile6.pdf]

**Supplement S6.**

*Descriptives and relations of all scales*

| # | Scale | *N* | *M* | *SD* | 1 | 2 | 3 | 4 | 5 | 6 | 7 | 8 | 9 | 10 | 11 | 12 | 13 | 14 |
|---|---|---|---|---|---|---|---|---|---|---|---|---|---|---|---|---|---|---|
| 1 | Reading Anxiety Trait | 269 | 10.320 | 6.465 | — | .429 | .317 | .348 | .470 | .443 | .403 | .362 | .334 | .349 | -.176 | -.055 | -.187 | -.244 |
| 2 | Reading Anxiety State 1 | 283 | 6.481 | 4.718 | .429 | — | .723 | .582 | .470 | .535 | .413 | .432 | .548 | .544 | -.274 | .118 | -.251 | -.311 |
| 3 | Reading Anxiety State 2 | 283 | 5.763 | 4.924 | .317 | .723 | — | .727 | .452 | .489 | .493 | .479 | .629 | .690 | -.177 | .102 | -.215 | -.218 |
| 4 | Reading Anxiety State 3 | 273 | 5.190 | 5.217 | .348 | .582 | .727 | — | .412 | .480 | .531 | .512 | .631 | .695 | -.219 | -.013 | -.245 | -.244 |
| 5 | Math Anxiety Trait | 260 | 9.854 | 7.625 | .470 | .470 | .452 | .412 | — | .369 | .345 | .324 | .437 | .403 | -.244 | .008 | -.221 | -.257 |
| 6 | Math Anxiety State 1 | 278 | 6.579 | 5.354 | .443 | .535 | .489 | .480 | .369 | — | .682 | .582 | .610 | .582 | -.052 | -.011 | -.134 | -.157 |
| 7 | Math Anxiety State 2 | 278 | 6.831 | 6.093 | .403 | .413 | .493 | .531 | .345 | .682 | — | .711 | .604 | .597 | .016 | .071 | -.150 | -.099 |
| 8 | Math Anxiety State 3 | 266 | 5.023 | 5.691 | .362 | .432 | .479 | .512 | .324 | .582 | .711 | — | .546 | .542 | -.059 | .034 | -.162 | -.133 |
| 9 | IQ Test Anxiety State 1 | 283 | 6.890 | 5.710 | .334 | .548 | .629 | .631 | .437 | .610 | .604 | .546 | — | .734 | -.111 | .024 | -.244 | -.248 |
| 10 | IQ Test Anxiety State 2 | 277 | 6.953 | 6.013 | .349 | .544 | .690 | .695 | .403 | .582 | .597 | .542 | .734 | — | -.087 | .105 | -.169 | -.200 |
| 11 | Reading Perf Time Press | 284 | -0.000 | 1.000 | -.176 | -.274 | -.177 | -.219 | -.244 | -.052 | .016 | -.059 | -.111 | -.087 | — | .177 | .465 | .514 |
| 12 | Reading Perf No Press | 277 | 0.000 | 1.000 | -.055 | .118 | .102 | -.013 | .008 | -.011 | .071 | .034 | .024 | .105 | .177 | — | .140 | .220 |
| 13 | Math Perf Time Press | 277 | -0.000 | 1.000 | -.187 | -.251 | -.215 | -.245 | -.221 | -.134 | -.150 | -.162 | -.244 | -.169 | .465 | .140 | — | .626 |
| 14 | Math Perf No Press | 271 | -0.000 | 1.000 | -.244 | -.311 | -.218 | -.244 | -.257 | -.157 | -.099 | -.133 | -.248 | -.200 | .514 | .220 | .626 | — |

*Note*. The reading and math performance scores were averaged from two standardized raw indicators, see text for further information.
